# High-Resolution Spectral Sleep Analysis Reveals a Novel Association Between Slow Oscillations and Memory Retention in Elderly Adults

**DOI:** 10.3389/fnagi.2020.540424

**Published:** 2021-01-11

**Authors:** Makoto Kawai, Logan D. Schneider, Omer Linkovski, Josh T. Jordan, Rosy Karna, Sophia Pirog, Isabelle Cotto, Casey Buck, William J. Giardino, Ruth O'Hara

**Affiliations:** ^1^Department of Psychiatry and Behavioral Sciences, School of Medicine, Stanford University, Stanford, CA, United States; ^2^Sierra Pacific Mental Illness Research Education and Clinical Centers (MIRECC), VA Palo Alto Health Care System, Palo Alto, CA, United States; ^3^Department of Psychology, Bar-Ilan University, Ramat-Gan, Israel; ^4^Department of Psychology, Dominican University of California, San Rafael, CA, United States

**Keywords:** overnight memory retention, aging, slow wave sleep, slow wave activity, slow oscillation

## Abstract

**Objective:** In recognition of the mixed associations between traditionally scored slow wave sleep and memory, we sought to explore the relationships between slow wave sleep, electroencephalographic (EEG) power spectra during sleep and overnight verbal memory retention in older adults.

**Design, Setting, Participants, and Measurements:** Participants were 101 adults without dementia (52% female, mean age 70.3 years). Delayed verbal memory was first tested in the evening prior to overnight polysomnography (PSG). The following morning, subjects were asked to recall as many items as possible from the same List (overnight memory retention; OMR). Partial correlation analyses examined the associations of delayed verbal memory and OMR with slow wave sleep (SWS) and two physiologic EEG slow wave activity (SWA) power spectral bands (0.5–1 Hz slow oscillations vs. 1–4 Hz delta activity).

**Results:** In subjects displaying SWS, SWS was associated with enhanced delayed verbal memory, but not with OMR. Interestingly, among participants that did not show SWS, OMR was significantly associated with a higher slow oscillation relative power, during NREM sleep in the first ultradian cycle, with medium effect size.

**Conclusions:** These findings suggest a complex relationship between SWS and memory and illustrate that even in the absence of scorable SWS, older adults demonstrate substantial slow wave activity. Further, these slow oscillations (0.5–1 Hz), in the first ultradian cycle, are positively associated with OMR, but only in those without SWS. Our findings raise the possibility that precise features of slow wave activity play key roles in maintaining memory function in healthy aging. Further, our results underscore that conventional methods of sleep evaluation may not be sufficiently sensitive to detect associations between SWA and memory in older adults.

## Introduction

Sleep is profoundly important for optimizing brain function and cognition. The rapidly advancing age of the global population highlights the urgent need to understand the dynamics between aging, sleep, and cognitive performance (Scullin and Bliwise, 2015). Of particular relevance for aging is the relationship between sleep and memory function (Diekelmann et al., 2009).

As early as 1924, Jenkins et al. demonstrated improved memory retention of acquired information after sleep compared to memory retention after an equivalent wake period (Jenkins and Dallenbach, 1924). Since the publication of these seminal findings, a growing body of research has demonstrated that the retention of verbal, visual, motor, and spatial information is improved after sleep (Plihal and Born, 1997; Walker et al., 2002, 2003; Tucker et al., 2006; Rasch and Born, 2007; Rasch et al., 2007; Lahl et al., 2008). The work of Wilson and McNaughton suggested the potential involvement of slow wave sleep (SWS) in memory formation (Wilson and McNaughton, 1994) when they observed re-expression of neuronal firing patterns in hippocampal circuits during SWS, similar to the hippocampal firing patterns observed during behavioral tasks preceding sleep (Wilson and McNaughton, 1994). Peigneux et al. observed positive associations between SWS, hippocampal activation, and recall performance in humans (Peigneux et al., 2004). Using functional magnetic resonance imaging, Takashima and colleagues suggested that the association of SWS with memory might reflect the transitioning of memories from the hippocampus to the ventral medial pre-frontal cortex (Takashima et al., 2006). Rasch et al. cued new memories during sleep by presenting an odor that had been presented as context during pre-sleep learning (Rasch et al., 2007) and found that re-exposure to the odor during SWS improved the retention of declarative memories (Rasch et al., 2007). The growing literature on SWS and memory underscores a potential role of SWS as integral to memory retention across a broad range of memory domains (Walker, 2009; Walker and Stickgold, 2010; Goerke et al., 2017).

Given that SWS, hippocampal volume, and episodic memory performance all decrease with age (Hasan and Broughton, 1994; Albert et al., 2001; Ohayon et al., 2004), the extant literature raises the question of whether poorer memory performance in older adults reflects declining SWS. Two important investigations, albeit with small sample sizes, found sleep to be associated with poorer memory performance in those at risk for dementia. Specifically, in amnestic mild cognitive impairment (aMCI), poorer subjective sleep is found to be associated with poorer memory performance (Westerberg et al., 2010). Furthering these findings, Westerberg et al. observed objective, macro- and microstructural sleep impairments–shorter SWS, as well as lower delta and theta power–in individuals with MCI, relative to cognitively normal controls (Westerberg et al., 2012). However, regardless of the level of cognitive impairment, increased delta and theta power were positively associated with overnight memory retention (Westerberg et al., 2012). To date, only a handful of additional published studies have examined memory in older adults before and after sleep. Of these publications, four lacked objective polysomnography (PSG) (Aly and Moscovitch, 2010; Wilson et al., 2012; Gudberg et al., 2015), two only evaluated the effect of naps on memory retention (Fogel et al., 2014; Baran et al., 2016), and only three examined nocturnal PSG and declarative memory (Rauchs et al., 2008; Mander et al., 2013; Scullin, 2013). A further complication is that while SWS is generally believed to decline with age (Ohayon et al., 2004), SWS depends on the manual scoring of sleep stages, and the variation of scoring interferes with the quantitative comparison between studies. Despite the advances in our understanding of age-related reduction of SWS in older adults, the relationships with OMR remain unclear due to the limited numbers of investigations, small sample sizes, lack of objective/PSG evaluation, variability in the memory measure employed and task difficulty, and physiologically incongruent definitions of SWS.

The consensus conclusion from this developing field is that associations between SWS and memory retention attenuate with age. However, Mander et al. identified a significant association between reduced slow wave activity (SWA) and poorer overnight memory retention (OMR) in a small sample of 15 older adults, suggesting a re-evaluation of this canonical framework. They did not, however, find any significant relationship between SWS and OMR. In that study, they used the term OMR (Mander et al., 2013), due to the different protocol design than the classical sleep-related memory consolidation experiments, in which the comparison of memory formation separated by wake period vs. sleep period in the same group was required.

Analyzing temporal characteristics of spectral power in the slow oscillation/delta range (0.5–4 Hz) provides a metric that more accurately captures the dynamics of the slow wave activity of sleep. Thus, here we aimed to address several longstanding issues and examine the polysomnographic features underlying OMR in older adults. We investigated the relationships between SWS and also spectral measures of slow wave activity, with pre-sleep delayed verbal memory performance, post-sleep OMR, and post-sleep delayed verbal memory on a new, alternate list. This design permitted us to address a number of prior limitations by including a large sample size and utilization of both traditional sleep staging and spectral-specific PSG analyses as they relate to OMR.

## Methods

### Study Design, Subject Characterization, and Sample Collection

Participants were 101 community-dwelling older adults enrolled in an investigation of sleep and cognitive function. This was a voluntary convenience sample recruited through advertisements and local senior centers from 2005 to 2010. The study protocol and consent were reviewed and approved by the Institutional Review Board, and informed consent was obtained from all participants. Initial evaluation included demographics, self-reported current and past medical status, the Mini-Mental State Exam (MMSE) to screen for dementia, and a Structured Clinical Interview for DSM-IV-TR (SCID-IV-TR) to screen for Axis I psychiatric disorders. Inclusion criteria were the following: being 50 years of age or over, the ability to give informed consent, and sufficient visual and auditory acuity for the cognitive testing. Individuals were excluded if they had an MMSE <26, a diagnosis of possible or probable dementia or a profile on the cognitive battery indicative of dementia, any serious medical illness, any Axis I disorder currently, or within the past 2 years on the SCID-IV-TR. Participants were also excluded if they were currently using a psychotropic medication, short-acting anxiolytics, sedative-hypnotics, medications with significant cholinergic or anticholinergic properties, or any FDA-approved medications for dementia.

### Procedures

#### Memory Assessment

A list-learning test was performed to examine delayed verbal memory directly prior to the assessment of PSG (List A). Participants were required to memorize a list of 16 words (8 abstracts; 8 concrete). We randomly assigned one of four lists, as designed by Yesavage et al. (1990). Four min of study time was allowed before the list was collected, and a 5-min, symbol-digit-distractor task was presented prior to delayed recall (Kraemer et al., 1983; Yesavage et al., 1990; O'Hara et al., 1998, 2007). After a delay of 5 min, participants were asked to recall and write down as many of the words as they could remember. The following morning, after a night of home PSG, participants were asked to recall and write down as many of the words from the original word list (List A) that they were asked to memorize the night before, without any re-presentation of List A. Then, the participants were randomly assigned one of the three remaining 16-word lists (List B). A similar procedure was employed: 4 min of study time, followed by a 5-min, symbol-digit-distractor task, and then recall. Every word recalled correctly was scored as a correct answer. All forms were counterbalanced across all conditions (Yesavage et al., 1990; O'Hara et al., 2007).

#### PSG and Sleep-Related Questionnaires

Ambulatory overnight PSG (Safiro Ambulatory PSG System; Compumedics, Charlotte, NC) was performed on all participants. Participants went to bed and arose according to their normal schedule. The contemporary, standard recording montage included scalp electroencephalography electrodes (C3, C4, O1, O2, M1, M2), chin electromyography, electro-oculography, electrocardiography, nasal pressure transducer, and oral airflow (thermistor), abdominal and thoracic respiratory-inductance plethysmography, finger pulse oximetry, and snoring audiography. All data were staged and scored by a registered polysomnography technologist and were reviewed by a certified sleep medicine physician. The strict 75 microvolt criterion to score slow wave sleep (stage 3 and stage 4 combined) were used following standard scoring guidelines (Rechtschaffen and Kales, 1968).

Sleep-related questionnaires included the Epworth sleepiness scale (ESS) to measure daytime sleepiness (Johns, 1991), the Pittsburgh Sleep Quality Index (PSQI) to assess recent sleep quality (Buysse et al., 1989), the Functional Outcomes of Sleep Questionnaire (FOSQ) to measure functional status including the impact of excessive sleepiness on multiple activities of daily living (Weaver et al., 1997), and the Morningness-Eveningness Questionnaire (MEQ) to assess chronotypes (Horne and Ostberg, 1976).

#### Quantitative Analysis of the Sleep EEG With an Automated Pipeline

Due to our prior observation of improved memory in association with increased Delta Activity at Sleep Onset (Kawai et al., 2016a,b), because the ratio of SWA is expected to be higher in the early stages of sleep (Carskadon and Dement, 2017), and because of variable total sleep times between individuals, the first ultradian cycle of NREM sleep was specifically examined, (a) as this is when SWA is most evident and, (b) to ensure comparability between individuals with respect to sleep duration. The spectral analysis was performed using the open-source spectral analysis software pipeline centered around the MATLAB App, SpectralTrainFig, available on the National Sleep Research Resource website (https://www.sleepdata.org/). The methods of artifact detection, additional human-assisted adjudication, and spectral analysis are described in detail in the recent article (Mariani et al., 2018). Analyses were performed using MATLAB R2017a (MathWorks, Inc., Natick, Massachusetts, USA). Using the developer-recommended analysis pipeline, the raw power spectrum was calculated on an epoch-by-epoch basis applying Welch's method (with ten, overlapping 4-s mini-epochs per 30-s epoch), with a 50% tapered cosine (Tukey) window (Welch, 1967; Harris, 1978). Power bands were defined as follows: Slow oscillation: 0.5–1 Hz, Delta: 1–4 Hz, Theta: 4–8 Hz, Alpha: 8–12 Hz, Sigma: 12–15 Hz, Slow sigma: 12–13.5 Hz, Fast sigma: 13.5–15 Hz, Beta: 15–20 Hz, and Total: 0–25 Hz. Using the protocols from the Mariani et al. paper, no EDF files were eliminated when applying these criteria. Any epochs with delta and beta artifact identified by the MATLAB software were also excluded from the analysis. There were a number of periods that were analyzed, all of which excluded any epochs scored as wake by the technician: (1) The period of sleep from sleep onset (SO) until the final epoch of sleep, (2) All epochs of NREM (N1, N2, and N3) sleep, (3) All epochs of REM sleep, and (4) The period identified as the approximate/aggregate first ultradian cycle of sleep (i.e., the first 88.5 min). Following removal of epochs visually scored as wake, absolute power (in μV^2^) and relative power (ratio of the absolute power in each frequency band over the absolute power of the total frequency band) were calculated in each 30-s epoch of sleep, for each of the bands of interest. Absolute and relative power were then calculated for NREM sleep period for the whole night and the first ultradian cycle.

#### Determination of First Ultradian Cycle

**Figure 1:**
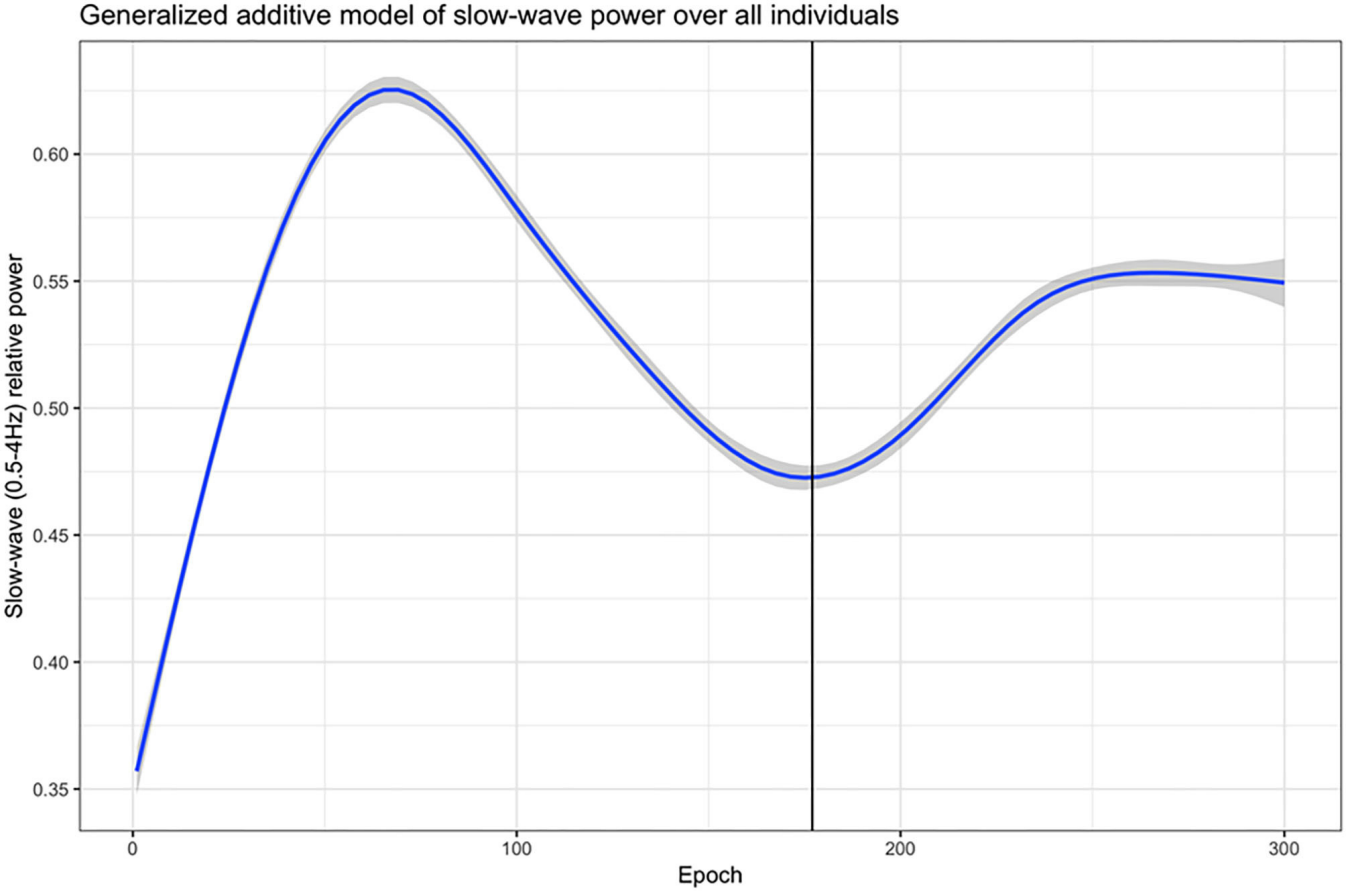
The first ultradian cycle of slow-wave activity (0.5–4 Hz band). A generalized additive model was used to generate a smoothed estimate of the relative slow-wave power (0.5–4 Hz band) progression for each individual over the first 300 epochs (150 min). The solid vertical line in the graph: The first post-elevation nadir of relative slow-wave power at 177 epochs (=88.5 min) in this cohort-wide model.

In order to define the first ultradian cycle of slow-wave activity, a generalized additive model was used to generate a smoothed estimate of the relative slow-wave power (0.5–4 Hz band) progression for each individual over the first 300 epochs (150 min)–a time span well-beyond the upper limit of the first ultradian cycle (120 min). Following this, another generalized additive model was used to approximate the end of the first ultradian cycle of slow-wave activity across the whole cohort. From this cohort-wide model, the epoch at which relative slow-wave power reached the first post-elevation nadir, 177 epochs (88.5 min), was defined as the end of the first ultradian cycle for the entire cohort, as this reflects the period with the greatest homeostatic sleep drive, as represented by slow-wave activity (Figure 1). We removed epochs scored as wake or REM during the first ultradian cycle for the analysis.

### Statistical Analysis

The mean and standard deviation of demographic measures, objective and subjective sleep parameters, memory scores, and retention rates were calculated and assessed for all 101 participants.

#### Primary Analysis

We employed partial correlation analyses to examine if SWS percentage predicted episodic memory performance and retention on the list-learning task adjusted for age, gender, and years of education. SWS served as the independent variable of interest, and performance on our list-learning measures of delayed verbal recall and overnight memory retention (OMR) served as dependent variables. Specifically, list-learning scores were calculated for each of three timepoints (a) pre-sleep/evening-administration, delayed-recall List A total score; (b) post-sleep/following-morning, delayed-recall performance on List A total score, without re-presentation of List A; (c) post-sleep/morning-administration, delayed-recall performance of alternate List B total score; and (d) overnight memory retention rate (OMR), which is defined as (b) divided by (a). Models were adjusted for age, gender, and years of education. We applied a Bonferroni correction for multiple comparisons (four parameters for primary analysis), setting alpha at 0.05/4=0.0125.

We also performed correlational analyses to examine the association of age with SWS. We repeated our primary analysis using spectral measures, specifically, absolute and relative power measures of slow wave activity as the independent variables of interest, and performance on our list-learning measures of delayed verbal recall and OMR as the dependent variables, as described above. We also performed correlational analyses to examine the association of age with spectral measures of slow wave activity.

#### Secondary Analyses

We noted that many of our participants did not have SWS (or N3) by the standard criteria (Rechtschaffen and Kales, 1968). Specifically, 76.4% (*n* = 78) did not exhibit SWS. Thus, we examined whole-night differences in spectral power between those who had SWS [SWS (+) group] and those without any SWS [SWS (–) group] by using two-tailed *t*-tests to compare absolute power and relative power in the frequency bands of interest. Additional SWS (+) and (–) comparisons were performed across all frequency bands of interest for the first ultradian cycle (88.5 min of sleep) and for all sleep following this first ultradian cycle.

Similar to the primary analyses, in the SWS (+) group and, independently, in the SWS (–) group, we performed partial correlation analyses (adjusted for age, gender, and years of education) to examine the association of our list-learning measures with absolute and relative power during and after the first 88.5 min of sleep. We also performed these analyses without adjustment for age, gender, or years of education.

For all partial correlation analyses, as interactive effects were included, all independent variables were centered at the median to ensure the interpretability of coefficients (Kraemer and Blasey, 2004). We applied a Bonferroni correction for multiple comparisons (four parameters for secondary analysis), setting alpha at 0.05/4 = 0.0125.

IBM SPSS Statistics for Macintosh, Version 25, Armonk, NY: IBM Corp. was used to perform all statistical analyses.

#### Exploratory Analyses

We performed correlational analyses between sleep measures or sleep questionnaires and delayed recall performance pre- or post-sleep or OMR. We also performed correlational analyses between slow oscillation and delta relative power in each sleep stage and OMR.

### Data Availability

Anonymized data not published within this article can be made available to qualified investigators upon request to the corresponding author.

## Results

### Demographics

Of the 101 participants, 53 (52.4%) were women, and the average age was 70.4 (SD 7.9 years; range 52–91). This was a generally well-educated (16.7 ± 2.7 years of education) and non-obese (BMI 27.7± 4.8 kg/m^2^) cohort (Table 1).

**Table 1:** Mean and standard deviation of demographic characteristics, PSG parameters, Sleep questionnaires, and list-learning scores in all 101 participants.

Demographics	Gender	Women = 53, Men = 48	
	Age	70.8 ± 7.9	years
	Years of Education	16.7 ± 2.7	years
	BMI	27.7 ± 4.8	kg/m^2^
PSG parameters	TST	344.3 ± 80.5	min
	TIB	509.7 ± 90.6	min
	N1	16.8 ± 10.3	%
	N2	68.9 ± 11.4	%
	N3 (=SWS)	1.4 ± 3.4	%
	REM	13 ± 5.9	%
	SL	37.8 ± 45.6	min
	WASO	125.4 ± 79.9	min
	SE	68.6 ± 15.5	%
	AHI	11.5 ± 12.9	events/h
	AvSpO2	93.1 ± 9.5	%
	MinSpO2	79.7 ± 18.9	%
	PLMI	8.3 ± 14.4	events/h
Sleep questionnaires	ESS	7.4 ± 4.3	
	PSQI	6.9 ± 3.9	
	FOSQ	17.9 ± 2.4	
	MEQ	58.7 ± 8.8	

*Numbers represent mean ± standard deviation, except gender, which showed the number of participants. BMI, body mass index (kg/m^2^); TST, total sleep time (minutes); TIB, time in bed (minutes); N1 to N3, Stage N1 to N3 sleep (%); SWS, slow wave sleep; REM, rapid eye movement sleep (%); SL, sleep latency (minutes); WASO, wake after sleep onset (minutes); SE, sleep efficiency (%); AHI, apnea hypopnea index (events/hour); AvSpO2, average oxygen saturation (%); MinSpO2, minimum oxygen saturation (%); PLMI, periodic limb movement index (events/hour); ESS, Epworth Sleepiness Scale; PSQI, Pittsburgh Sleep Quality Index; FOSQ, Functional Outcomes of Sleep Questionnaire; MEQ, Morningness-Eveningness Questionnaire*.

### Sleep Parameters

The mean and SD for total sleep time (TST) (minutes), time in bed (TIB) (minutes), sleep stages (%), sleep onset latency (minutes), WASO (minutes), and sleep efficiency (%) are provided in Table 1. The average distribution of sleep stages was within the range expected for a single-night sleep study in an elderly population (Table 1).

### Memory Recall and Retention

The mean and SD for the list-learning memory total recall scores for each of the three memory test times–List A (evening administration and recall prior to sleep), List A (delayed recall only of List A the morning after sleep, there was no re-presentation of List A), and List B (following List A recall the morning after sleep, List B, an alternate form, was administered and recall assessed), and OMR are presented in Table 2.

**Table 2:** Mean, standard deviation, and partial correlation analysis to examine the association of SWS and list-learning scores covaried for age, gender, and year of education and correlation coefficient.

**All 101 participants, partial correlation with SWS, covaried for age, gender, years of education**			
	**Mean and SD**	**Correlation**	***p***
Evening 5-min recall List A	6.7 ± 3.6	0.153	0.132
Overnight morning recall List A	4.8 ± 3.7	0.231	0.023
Morning 5-min recall with alt List B	6.9 ± 3.9	0.168	0.098
OMR	0.7 ± 0.4	0.093	0.37

*SD, Standard deviation; SWS, slow wave sleep (%)*.

*Evening 5-min recall List A: Pre-sleep evening delayed recall List A total score prior to PSG*.

*Overnight morning recall List A: Post-sleep/following-morning, delayed-recall performance on List A total score*.

*Morning 5-min recall alt List B: Post-sleep/morning-administered, delayed-recall performance on alternate List B total score*.

*OMR: Overnight memory retention, defined as post-sleep/following-morning, delayed-recall performance on List A total score divided by pre-sleep evening delayed-recall performance on List A total score*.

### Primary Analyses

We conducted partial correlation analyses using our continuous measure of technician-scored SWS as our predictor, with total verbal-memory recall, pre- and post-sleep, and retention as our dependent measures. We found that SWS was not significantly associated with pre-sleep/evening, delayed recall on List A, total delayed-recall performance on List A/post-sleep, nor with post-sleep/morning-administered, delayed-recall performance on alternate List B. SWS was not significantly associated with OMR following Bonferonni correction (Table 2).

#### Spectral Analysis

In all participants, the partial correlation analysis, covaried for age, gender, and years of education, found no significant correlation between OMR and absolute and relative measures of SWA (0.5–4 Hz, combining slow oscillation and the delta band) during the NREM sleep period for the whole night. As mentioned previously, since the duration of sleep is highly variable, to better control for this, we focused on power spectral analysis of NREM sleep during the first 177 epochs (88.5 min) after sleep onset, when participants finished the first ultradian cycle of SWA (0.5–4 Hz, combining slow oscillation and the delta band). In all participants, the partial correlation analysis, covaried for age, gender, and years of education, did not show a significant correlation between the slow-oscillation relative power during the first ultradian cycle and OMR after Bonferroni correction (Supplementary Table 2).

In our cohort, age and SWS were not correlated by Spearman's test, and absolute and relative power measures of SWA were also not correlated with age.

### Secondary Analyses

#### Groups With or Without SWS

As mentioned above, we noted that many of our participants did not have SWS scored by the standard criteria (Rechtschaffen and Kales, 1968). Specifically, 76.2% (*n* = 77) did not exhibit SWS. Among the remaining sleep parameters, no statistically significant difference was found between the SWS (+) and (–) groups. Post-sleep/following-morning, delayed-recall performance on List A total was significantly higher in the SWS (+) group (6.3 ± 3.9 vs. 4.4 ± 3.5, *t*(99) = 2.27, *p* = 0.025, d = 0.51) with medium effect size (Table 3). However, this difference was not significant following Bonferroni correction. The unadjusted analysis did not change the finding (Supplementary Table 3).

**Table 3:** Comparison in list-learning scores between 24 participants with and 78 without any SWS.

	**SWS (+) n=24**	**SWS (–) n=77**	***p***	**Cohen's *d***
Evening 5-min recall	7.9 ± 3.6	6.3 ± 3.6	0.066	0.44
Overnight morning recall	6.3 ± 3.9	4.4 ± 3.5	0.025	0.51
Morning 5-min recall with alt list	7.4 ± 4.7	6.8 ± 3.6	0.493	0.14
OMR	0.75 ± 0.2	0.64 ± 0.4	0.245	0.32

*SWS, slow wave sleep (%)*.

*Evening 5-min recall List A: Pre-sleep evening delayed recall List A total score prior to PSG*.

*Overnight morning recall List A: Post-sleep/following-morning, delayed-recall performance on List A total score*.

*Morning 5-min recall alt List B: Post-sleep/morning-administered, delayed-recall performance on alternate List B total score*.

*OMR: Overnight memory retention, defined as post-sleep/following-morning, delayed-recall performance on List A total score divided by pre-sleep evening delayed-recall performance on List A total score*.

Despite the apparent lack of scorable SWS, the normal distribution of slow-oscillation relative power during the first 88.5 min of sleep suggests that slow-oscillation relative power may be an important measure of slow-wave activity in older adults who don't exhibit SWS. Thus, we examined the association of slow-oscillation relative power (0.5–1 Hz) and our memory outcomes in secondary analyses.

**Figure 2:**
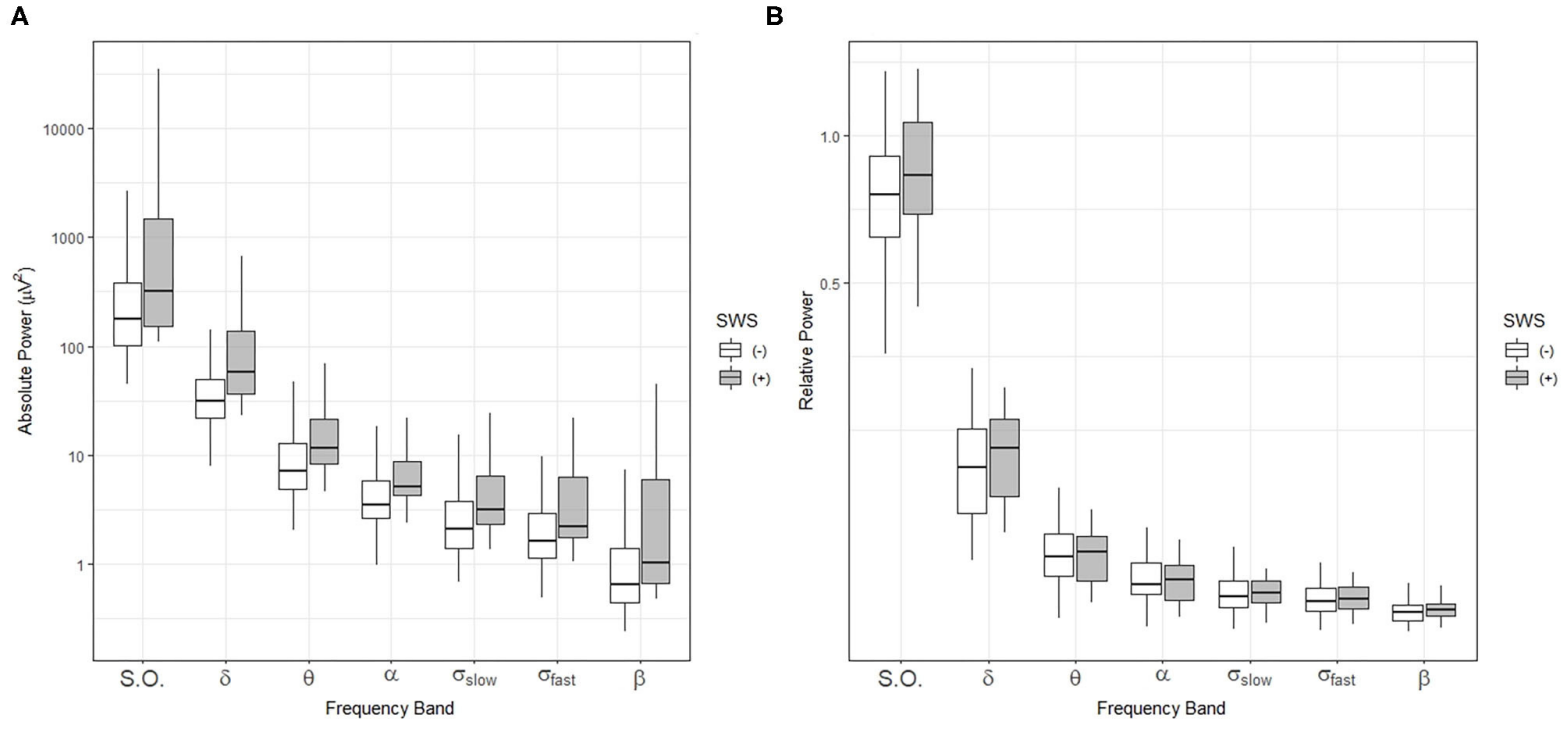
Comparisons of sleep-related frequency bands over the entire sleep period in individuals with (+) and without (–) slow-wave sleep (SWS). Box-and-whisker plot of absolute power **(A)** and relative power **(B)** during NREM sleep for the whole night. SWS (+): participants with slow wave sleep, SWS (–): participants without slow wave sleep, S.O.: slow oscillation (0.5–1 Hz), δ: delta (1–4 Hz), θ: theta (4–8 Hz), α: alpha (8–12 Hz), σ_slow_: slow sigma (12–13.5 Hz), σ_fast_: fast sigma: (13.5–15 Hz), β: beta: (15–20 Hz).

Thus, we performed spectral analysis of the sleep EEG signal and compared the absolute and relative power of sleep-related frequency bands between the SWS (+) and (–) groups (Figures 2A,B). Absolute and relative power in all power bands during the NREM sleep period for the whole night did not show a statistically significant difference (Figures 2A,B).

**Figure 3:**
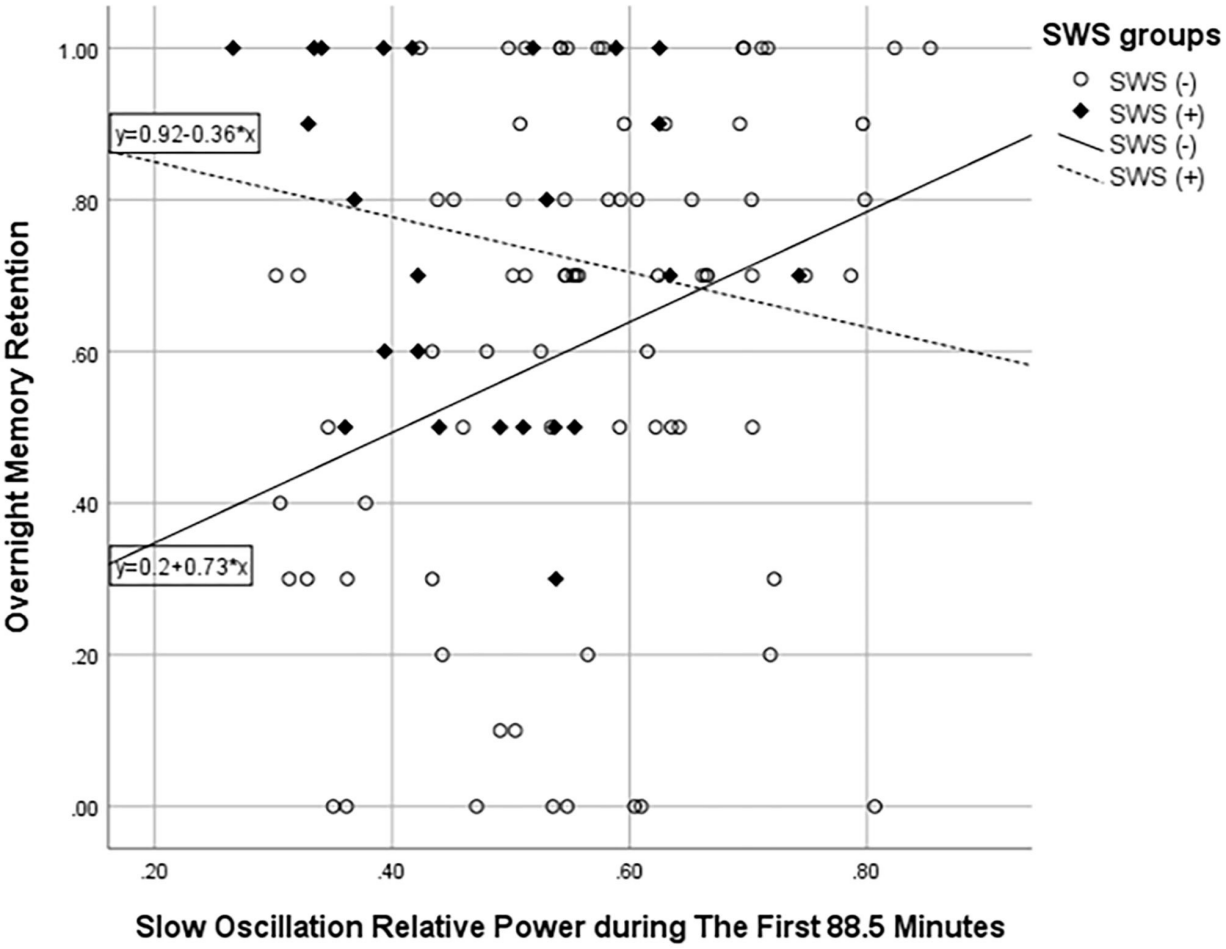
Scatter plot showing the association between slow oscillation relative power during NREM sleep in the first 88.5 min and overnight memory retention. ° represents the SWS (–) group and ♦ SWS (+) group.

**Figure 4:**
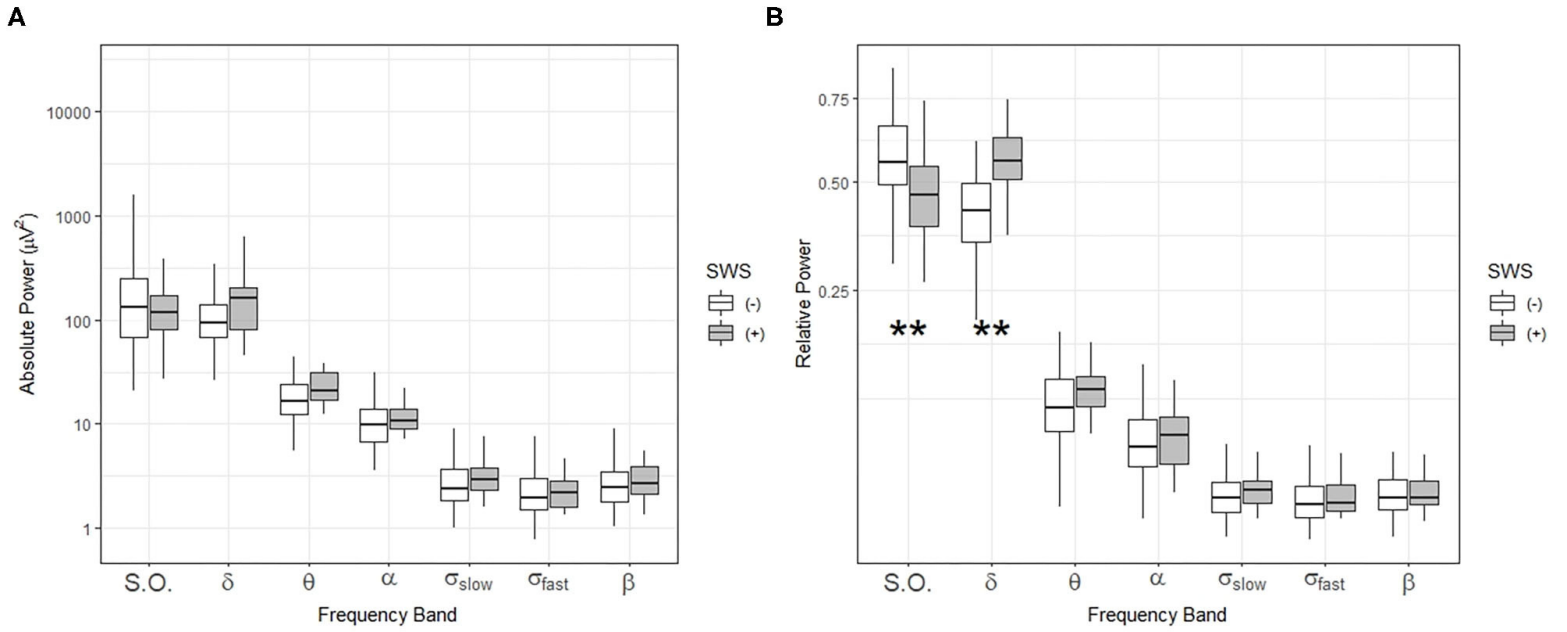
Comparisons of sleep-related frequency bands during NREM sleep in the first ultradian cycle (88.5 min) in individuals with (+) and without (–) slow-wave sleep (SWS). Box-and-whisker plot of absolute power **(A)** and relative power **(B)** during the first 88.5 min of sleep. SWS (+): participants with slow wave sleep, SWS (–): participants without slow wave sleep. Centerline, median; box limits, upper and lower quartiles; whiskers, 1.5x interquartile range. S.O.: slow oscillation (0.5–1 Hz), δ: delta (1–4 Hz), θ: theta (4–8 Hz), α: alpha (8–12 Hz), σ_slow_: slow sigma (12–13.5 Hz), σ_fast_: fast sigma: (13.5–15 Hz), β: beta: (15–20 Hz). **p < 0.01 by *t*-test.

Then, we compared absolute and relative power for the SWS (–) group with the SWS (+) group during NREM sleep in the first ultradian cycle of sleep. In the SWS (–) group only, partial correlation analyses, adjusting for age, gender, and years of education, found that slow-oscillation relative power (0.5–1 Hz) during NREM sleep period in the first 88.5 min is positively associated with higher OMR, following Bonferroni correction for multiple testing (*r* = 0.335, *p* = 0.003) (Table 4). Our findings did not change with unadjusted analysis (Supplementary Table 4). Homogeneity of correlation tests showed that the difference of correlation of the slow-oscillation relative power (0.5–1 Hz) during the first 88.5 min and OMR is significantly different between the SWS (–) and (+) groups (Chi-square= 8.28, *p* = 0.004) (Figure 3). We also found significantly higher slow oscillation relative power in SWS (–) group than SWS (+) (Cohen's d = 0.72, *t*(99) = 2.972, *p* = 0.004) (Figures 4A,B). As such, it is notable that, despite the lack of SWS, participants in the SWS (–) group still have a wide range of normally distributed relative power in the slow oscillation band during the first 88.5 min of sleep.

**Table 4:** Partial correlation analysis to examine the association of slow oscillation (0.5–1 Hz) during the first 88.5 min of sleep and list-learning scores covaried for age, gender, and year of education among 90 participants without SWS and correlation coefficient.

**77 participants without SWS, partial correlation with relative power spectrum of slow oscillaition during the first 88.5 min (177 epochs), covaried for age, gender, years of education**		
	**Correlation**	***p***
Evening 5-min recall List A	0.092	0.434
Overnight morning recall List A	0.257	0.027
Morning 5-min recall with alt List B	0.032	0.789
OMR	0.335	0.003^*^

* *Statistically significant after Bonferroni correction*.

*Evening 5-min recall List A: Pre-sleep evening delayed recall List A total score prior to PSG*.

*Overnight morning recall List A: Post-sleep/following-morning, delayed-recall performance on List A total score*.

*Morning 5-min recall alt List B: Post-sleep/morning-administered, delayed-recall performance on alternate List B total score*.

*OMR: Overnight memory retention, defined as post-sleep/following-morning, delayed-recall performance on List A total score divided by pre-sleep evening delayed-recall performance on List A total score*.

In comparison to the SWS (–) group, for the SWS (+) group, the relative power spectrum of slow oscillation (0.5–1 Hz) or delta band (1–4 Hz) during the first 88.5 min of sleep was not significantly associated with any memory performance measures.

Additionally, in analyses examining the remainder of the sleep period following the first ultradian cycle (i.e., all sleep after the first 88.5 min), no frequency bands were associated with memory performance measures in either the SWS (–) or the SWS (+) group.

Age does not differ between the SWS (–) and (+) groups by *t*-test (*t* = 1.12, *p* = 0.267). Further, the findings of a statistically significant association of SO and OMR in SWS (–) but not in the full sample nor in the SWS (+) group, did not change when we covaried by age.

#### Exploratory Analyses

The association of all other sleep parameters with OMR were examined in exploratory analyses. None of our other sleep measures or sleep questionnaires were associated with delayed recall performance pre- or post-sleep or with OMR. We also did not find any statistically significant correlation between slow oscillation and delta relative power in each sleep stage and OMR.

## Discussion

Our study, which is one of the largest investigations of its kind, provides novel insights into the relationship of objective, overnight sleep EEG, and overnight memory retention (OMR) in community-dwelling, typically aging, older adults. In our research, we did not observe any statistically significant association of OMR with SWS, which is in line with previous studies (Rauchs et al., 2008; Mander et al., 2013; Scullin, 2013). Backhaus et al., on the other hand, reported a positive correlation of SWS to OMR (Backhaus et al., 2007), but examined individuals aged 48–55, who are younger than our own and other investigations of sleep and OMR in older adults.

With respect to electroencephalographic (EEG) power spectra during sleep, we found a statistically significant positive association of slow-oscillation relative power (0.5–1 Hz) in the first ultradian cycle with OMR in the SWS (–) group. This is in line with another investigation that employed overnight assessment of slow wave activity. Focusing on the power spectra of SWA (0.8–4.6 Hz), in 15 older adults, Mander et al. found a reduction of SWA (0.8–4.6 Hz) in NREM sleep was associated with worse declarative memory and poorer overnight retention (Mander et al., 2013). Similarly, Varga et al. also reported a positive association between slow wave activity (0.5–4 Hz) and a spatial navigational memory consolidation task in 13 older adults (Varga et al., 2016). It is noteworthy that similar to our own investigation, Mander et al. (2013), did not observe a statistically significant association between SWS and OMR.

In another investigation, Anderson et al. found slow oscillation (0.5–1 Hz) in the first NREM sleep period to be associated with non-verbal planning and verbal fluency in 24 older adults with an age range of 61–75, but these cognitive functions were assessed on a separate day, and the study did not measure OMR (Anderson and Horne, 2003).

In our study, we also observed that large numbers of sleep studies showed an absence of SWS, which does not mean there is a lack of SWA. Indeed, we found those without SWS retained a normally distributed range of slow oscillation (0.5–1 Hz) activity. Building upon this, in secondary analyses, we found that among those with no SWS, slow-oscillation (0.5–1 Hz) relative power in the first ultradian cycle was positively associated with OMR. This is not unexpected, given that the first 88.5 min of sleep approximates the completion of the first ultradian sleep cycle of slow wave activity calculated from our older-adult cohort. This is because the first ultradian cycle of sleep is when the homeostatic sleep drive is most profound and is known to have more SWA than subsequent ultradian cycles of sleep (Carskadon and Dement, 2017).

During NREM sleep in the first ultradian cycle, we found that relative power comparisons demonstrated that the SWS (–) group actually had significantly higher relative power in the slow oscillation band (0.5–1 Hz) compared to the SWS (+) group. No significant differences were observed between the two groups with respect to any other frequency bands during or after the first 88.5 min of sleep.

Interestingly, Westerberg et al. (2012), observed increased delta power to be positively associated with overnight memory retention in both MCI and healthy participants (Westerberg et al., 2012); their delta power range (0.5–4.5 Hz) includes the slow oscillation power range (0.5–1 Hz) we observed to be associated with OMR in our study.

Regarding the limitation of SWS as a sleep parameter predicting memory function in older adults, Scullin et al. reported a lower percent of SWS in older adults compared to young adults; however, there was no apparent association of SWS with OMR (Scullin, 2013). In other studies investigating the effect of SWS on the daytime memory function in older adults, the positive effect of SWS diminishes as well. Spiegel et al. reported no association between SWS by conventional scoring and memory measures in older adults (Spiegel et al., 1999). A larger study of 2,601 community-dwelling older men, investigating sleep stages and cognitive function, also did not find an association between SWS and trail making or Mini-Mental State Examination scores (Song et al., 2015). None of these investigations employed spectral analyses that capture the slow wave activity missed by the amplitude cutoffs imposed by the standard scoring criteria for slow wave sleep. Studies such as ours, Westerberg, and that of Mander et al. find that such spectral measures of slow wave activity are associated with memory in older adults, underscoring the importance of spectral analyses for understanding sleep's role in memory function and decline in the elderly.

With respect to the age-related effect on SWS and SWA, Redline et al. reported in their cohort, age range, 37–92 that SWS was significantly lower in those older than 54 compared with younger individuals (Redline et al., 2004). Sprecher et al. reported an age-related reduction of SWA in a younger cohort that encompassed younger, older adults (18–65) (Sprecher et al., 2016). In our own investigation, which had covered a wide age range (52–91), contrary to previous reports, we did not find a significant effect of age on SWS or SWA. The lack of age effect in our cohort may be because the age-related reduction of SWS and SWA has already occurred in most participants in our cohort. Future studies should examine this issue more fully.

Given our lack of statistically significant association between age, SWS, and SWA, it appears that our main finding of an association between slow oscillation and OMR in the total sample and the SWS (–) group is not driven by age.

Our findings suggest that, despite the general attenuation of readily discernable slow wave activity in the delta band (1–4 Hz), during the period of greatest slow-wave activity–the first ultradian cycle–there is the preservation of an ultra-low-frequency oscillator (0.5–1 Hz) in older adults, among those with *no* SWS. Most importantly, in this SWS (–) group only, we found a statistically significant positive association of slow-oscillation relative power (0.5–1 Hz) with OMR (Figure 3) with a medium effect size (Table 4); whereas, this association with OMR was not evident for relative delta power (1–4 Hz) in the SWS (–) group.

Comparatively, we did not see this association for the SWS (+) group (Figure 3), nor for any frequency band for sleep after the first 88.5 min. This observation that slow oscillation is associated with OMR is in line with a previous study demonstrating a positive association of slow oscillation (0.5–1 Hz) in the first NREM sleep period with better daytime frontal cognitive function in older adults, albeit assessed on a separate day (Anderson and Horne, 2003).

This leaves the question as to why we observed no impact of slow oscillation on the memory and OMR of those who had SWS. It is notable that participants with preserved SWS had better overall memory performance the morning after sleep, but not significantly more memory retention than was observed in the SWS negative group. This may reflect the fact that they have less opportunity for retention, as they may have already potentially reached the asymptote of their memory performance. The lack of a significant correlation between slow oscillation and OMR could occur because of a ceiling effect in the group with retained SWS, with low variation in over-night recall performance and slow-oscillation relative power. Of note, we did not observe this association in those with both SWS and lower pre-sleep memory scores, suggesting that a ceiling effect was not at play. However, we cannot completely exclude this possibility due to the lack of power from a reduced sample size in this subgroup analysis.

OMR could also vary with the amount of learning pre-sleep, in that very low rates of evening learning might not yield memories strong enough to result in OMR. To examine this possibility, we conducted *post-hoc* analyses, examining the correlation of OMR with SWS after covarying out the level of performance on the evening test. We also performed the analysis using a higher and lower evening score group by a median split to see if SWS was positively correlated with OMR in the higher recall group. Neither analysis yield statistically significant results.

Another possibility is that subjects who are more sleep-deprived may do poorly at evening recall but then have more SWS because of their sleep deprivation, thus doing better on the recall test in the morning due to being well-rested. We performed a *post-hoc* correlational analysis with ESS, which was obtained in the evening before the sleep study, with OMR, evening scores, and SWS. No statistically significant correlations were found, suggesting that this explanation does not account for the lack of the observed association of slow oscillation on the memory and OMR in the SWS (+) group.

The mechanism by which slow oscillation may contribute to enhanced memory retention in older adults is largely unknown. However, it is reported that slow oscillation is generated and sustained by the cerebral cortex in cats (Steriade et al., 1993; Timofeev and Steriade, 1996; Timofeev et al., 2000) and *in vitro* (Shu et al., 2003) and is disrupted by disconnection of intracortical synaptic linkages in cats (Amzica and Steriade, 1995). Helfrich et al. reported a positive association of the coupling between slow oscillation and the sleep spindle with OMR (Helfrich et al., 2018). They reported this coupling to be more impaired in older than younger adults (Helfrich et al., 2018). In this investigation, we did not have access to sleep spindles but will examine this issue in our future investigations.

High-density EEG recording indicates that slow oscillations provide a fundamental blueprint of cortical excitability and connectivity during sleep (Massimini et al., 2004). Potential explanations for our observation of a positive association of slow oscillation with OMR, which occurred only in individuals lacking SWS, include: (1) a potential compensatory mechanism of slow oscillations, in the setting of age-related reductions of SWS; (2) a residual slow oscillation component of vanishing SWS, and (3) breach of the fundamental pacemaker rhythm representing diffuse cortical connectivity.

Recently, interventions with acoustic stimulation, transcranial direct current stimulation (tDCS), and pharmacological interventions have aimed to enhance slow wave activity during sleep (Zhang and Gruber, 2019). tDCS showed improvement in SWA and overnight memory retention in young adults (Marshall et al., 2004, 2006). Some studies demonstrated enhanced slow oscillation (<1 Hz) with improved memory (Westerberg et al., 2015; Ladenbauer et al., 2016). Studies utilizing acoustic stimulation methods during sleep can increase SWA, especially slow oscillation (<1 Hz), and improve sleep-dependent memory retention both in young (Ngo et al., 2013, 2015; Kawai et al., 2016a; Leminen et al., 2017; Papalambros et al., 2017; Ong et al., 2018) and older adults (Papalambros et al., 2017). Pharmacological trials with sodium oxybate show mixed results on cognitive function. Walsh et al. reported that sodium oxybate increased slow wave activity and better psychomotor vigilance test in healthy young adults (Walsh et al., 2010). On the other hand, Vienne et al. reported that an increased level of delta power after administration of sodium oxybate did not improve memory retention in the nap study (Vienne et al., 2012).

Together these studies suggest that slow oscillations likely play a role in memory retention and, for the most part, are in line with the finding of our study in older adults. Our findings suggest the involvement of more precise features of sleep, which can guide future stimulation investigations, in terms of both the temporal and frequency domains that can be targeted in order to optimize improvements in sleep-associated cognitive function. Further, future investigations should investigate the relationship of slow oscillations to a broader range of cognitive functions and examine if any associated improvements are maintained over time.

To further investigate the mechanism of the effect of slow oscillation on memory, future studies should also consider integrating K-complex or sleep spindle coupling to examine if the effect of slow oscillation on OMR is independent of these components.

Further, there is tremendous variability across studies of sleep and OMR, in terms of the memory measure employed, with some investigations using neuropsychological measures, some utilizing paired-associate tests, and others measuring memory recognition and not recall. There is also variability among these studies with respect to the difficulty of the memory measures assessed. Future studies in this field should consider a broader range of memory tasks when examining sleep and OMR to determine if this relationship varies according to the memory task employed.

Limitations of our study include: (1) the cross-sectional design, which limits the ability to explore mediational relationships of SWS or slow oscillations on cognitive decline or OMR decline in older adults, (2) a lack of frontal EEG electrodes, due to utilizing traditional PSG montage before the issuance of the 2007 AASM polysomnography guidelines (Ruehland et al., 2011), (3) a lack of comparison of the memory retention effect with an intervening period of the wake in the daytime and possible circadian confound, (4) the OMR measure was obtained through a division calculation with two measures (evening and morning scores), which may have magnified variability due to extraneous factors, even though we believe the alternative calculation of the subtraction of two numbers (pre, and post-sleep) is inaccurate, especially in those who scored low in pre-sleep test, (5) a lack of data collection over two nights to avoid first-night effects or sufficient information regarding habitual sleep duration, (6) utilizing a single memory measure for the memory domain of interest, (7) the lack of young, comparison group, and (8) lack of neuroimaging or high-density EEG recording to examine the topographical associations of the slow-oscillatory activity. To mitigate the limitations listed above, we tried several approaches including, (1) analyzing relative power of SWA to minimize the disadvantage of lack of frontal EEG electrodes, (2) using the term of OMR to clarify the difference from consolidation research, (3) measuring delayed memory recall from list B in the morning to investigate the circadian effect on memory, and (4) using ambulatory PSG at home instead of in-lab PSG to minimize the first-night effect.

In summary, our findings suggest a complex relationship between SWS, SWA, and OMR, with increased levels of SWS associated with better delayed recall performance overall, but not with overnight memory retention. Similarly, spectral power across the full night period was also not associated with overnight memory retention. However, our investigation found that slow oscillation (0.5–1 Hz) during the first ultradian sleep cycle of slow wave activity is key for overnight memory retention in older adults, specifically in those lacking SWS, even though there is still uncertainty regarding the exact mechanism subserving this finding. Further, our findings demonstrate the potential importance of temporal and frequency features of sleep for OMR. Finally, our study underscores that conventional methods of sleep evaluation, such as scoring of N3 sleep/SWS, may not be sufficient or sensitive enough for detecting the association of slow wave activity and memory in older adults.

## Data Availability Statement

All datasets generated for this study are included in the article/Supplementary Material.

## Ethics Statement

The studies involving human participants were reviewed and approved by Stanford University Internal Review Board. The patients/participants provided their written informed consent to participate in this study.

## Author Contributions

MK, LS, and RO'H contributed to the conception and design of the study, and were responsible for the writing of the manuscript and the generation of figures. All authors contributed to the acquisition and analysis of data.

## Conflict of Interest

The authors declare that the research was conducted in the absence of any commercial or financial relationships that could be construed as a potential conflict of interest.

## References

[B1] Albert M. S. Moss M. B. Tanzi R. Jones K. (2001). Preclinical prediction of AD using neuropsychological tests. J. Int. Neuropsychol. Soc. 7, 631–639. 10.1017/S1355617701755105 11459114

[B2] Aly M. Moscovitch M. (2010). The effects of sleep on episodic memory in older and younger adults. Memory 18, 327–334. 10.1080/09658211003601548 20182945

[B3] Amzica F. Steriade M. (1995). Disconnection of intracortical synaptic linkages disrupts synchronization of a slow oscillation. J. Neurosci. 15, 4658–4677. 10.1523/JNEUROSCI.15-06-04658.1995 7790931 PMC6577695

[B4] Anderson C. Horne J. A. (2003). Prefrontal cortex: links between low frequency delta EEG in sleep and neuropsychological performance in healthy, older people. Psychophysiology 40, 349–357. 10.1111/1469-8986.00038 12946109

[B5] Backhaus J. Born J. Hoeckesfeld R. Fokuhl S. Hohagen F. Junghanns K. (2007). Midlife decline in declarative memory consolidation is correlated with a decline in slow wave sleep. Learn. Memory 14, 336–341. 10.1101/lm.470507 PMC1876757 17522024

[B6] Baran B. Mantua J. Spencer R. M. (2016). Age-related changes in the sleep-dependent reorganization of declarative memories. J. Cogn. Neurosci. 28, 1–11. 10.1162/jocn_a_00938 26918588 PMC6419091

[B7] Buysse D. J. Reynolds C. F. III. Monk T. H. Berman S. R. Kupfer D. J. (1989). The Pittsburgh Sleep Quality Index: a new instrument for psychiatric practice and research. Psychiatry Res. 28, 193–213. 10.1016/0165-1781(89)90047-4 2748771

[B8] Carskadon M. A. Dement W. C. (2017). Normal Human Sleep: An Overview, in Principles and Practice of Sleep Medicine, 6th Edn, eds. Kryger M. Roth T. Dement W. C. (Philadelphia, PA: Elsevier). 10.1016/B978-1-4160-6645-3.00002-5

[B9] Diekelmann S. Wilhelm I. Born J. (2009). The whats and whens of sleep-dependent memory consolidation. Sleep Med. Rev. 13, 309–321. 10.1016/j.smrv.2008.08.002 19251443

[B10] Fogel S. M. Albouy G. Vien C. Popovicci R. King B. R. Hoge R.. (2014). fMRI and sleep correlates of the age-related impairment in motor memory consolidation. Hum. Brain Mapp. 35, 3625–3645. 10.1002/hbm.22426 24302373 PMC6869653

[B11] Goerke M. Müller N. G. Cohrs S. (2017). Sleep-dependent memory consolidation and its implications for psychiatry. J. Neural Transm. 124, 163–178. 10.1007/s00702-015-1476-3 26518213

[B12] Gudberg C. Wulff K. Johansen-Berg H. (2015). Sleep-dependent motor memory consolidation in older adults depends on task demands. Neurobiol. Aging 36, 1409–1416. 10.1016/j.neurobiolaging.2014.12.014 25618616 PMC4353561

[B13] Harris F. J. (1978). On the use of windows for harmonic analysis with the discrete Fourier transform. Proc. IEEE 66, 51–83. 10.1109/PROC.1978.10837

[B14] Hasan J. Broughton R. (1994). Quantitative Topographic EEG Mapping During Drowsiness and Sleep Onset, in Sleep Onset: Normal and Abnormal Processes, 1st Edn, eds Ogilvie R. D. Harsh J. R. (Washington, DC: American Psychological Association). 10.1037/10166-013

[B15] Helfrich R. F. Mander B. A. Jagust W. J. Knight R. T. Walker M. P. (2018). Old brains come uncoupled in sleep: slow wave-spindle synchrony, brain atrophy, and forgetting. Neuron 97, 221–230. 10.1016/j.neuron.2017.11.020 29249289 PMC5754239

[B16] Horne J. A. Ostberg O. (1976). A self-assessment questionnaire to determine morningness-eveningness in human circadian rhythms. Int. J. Chronobiol. 4, 97–110. 1027738

[B17] Jenkins J. G. Dallenbach K. M. (1924). Obliviscence during Sleep and Waking. Am. J. Psychol. 35, 605–612. 10.2307/1414040

[B18] Johns M. W. (1991). A new method for measuring daytime sleepiness: the Epworth sleepiness scale. Sleep 14, 540–545. 10.1093/sleep/14.6.540 1798888

[B19] Kawai M. Beaudreau S. A. Gould C. E. Hantke N. C. Cotto I. Jordan J. T.. (2016a). Longitudinal association of delta activity at sleep onset with cognitive and affective function in community-dwelling older adults. Int. J. Geriatr. Psychiatry 31, 1124–1135. 10.1002/gps.4554 27554208

[B20] Kawai M. Beaudreau S. A. Gould C. E. Hantke N. C. Jordan J. T. O'Hara R. (2016b). Delta activity at sleep onset and cognitive performance in community-dwelling older adults. Sleep 39, 907–914. 10.5665/sleep.5652 26943464 PMC4791624

[B21] Kraemer H. C. Blasey C. M. (2004). Centring in regression analyses: a strategy to prevent errors in statistical inference. Int. J. Methods Psychiatr. Res. 13, 141–151. 10.1002/mpr.170 15297898 PMC6878533

[B22] Kraemer H. C. Peabody C. A. Tinklenberg J. R. Yesavage J. A. (1983). Mathematical and empirical development of a test of memory for clinical and research use. Psychol. Bull. 94:367 10.1037/0033-2909.94.2.367

[B23] Ladenbauer J. Külzow N. Passmann S. Antonenko D. Grittner U. Tamm S.. (2016). Brain stimulation during an afternoon nap boosts slow oscillatory activity and memory consolidation in older adults. Neuroimage 142, 311–323. 10.1016/j.neuroimage.2016.06.057 27381076

[B24] Lahl O. Wispel C. Willigens B. Pietrowsky R. (2008). An ultra short episode of sleep is sufficient to promote declarative memory performance. J. Sleep Res. 17, 3–10. 10.1111/j.1365-2869.2008.00622.x 18275549

[B25] Leminen M. M. Virkkala J. Saure E. Paajanen T. Zee P. C. Santostasi G.. (2017). Enhanced memory consolidation via automatic sound stimulation during non-REM sleep. Sleep 40:zsx003. 10.1093/sleep/zsx003 28364428 PMC5806588

[B26] Mander B. A. Rao V. Lu B. Saletin J. M. Lindquist J. R. Ancoli-Israel S.. (2013). Prefrontal atrophy, disrupted NREM slow waves and impaired hippocampal-dependent memory in aging. Nat. Neurosci. 16, 357–364. 10.1038/nn.3324 23354332 PMC4286370

[B27] Mariani S. Tarokh L. Djonlagic I. Cade B. E. Morrical M. G. Yaffe K.. (2018). Evaluation of an automated pipeline for large-scale EEG spectral analysis: the National Sleep Research Resource. Sleep Med. 47, 126–136. 10.1016/j.sleep.2017.11.1128 29803181 PMC5976521

[B28] Marshall L. Helgadóttir H. Mölle M. Born J. (2006). Boosting slow oscillations during sleep potentiates memory. Nature 444:610. 10.1038/nature05278 17086200

[B29] Marshall L. Mölle M. Hallschmid M. Born J. (2004). Transcranial direct current stimulation during sleep improves declarative memory. J. Neurosci. 24, 9985–9992. 10.1523/JNEUROSCI.2725-04.2004 15525784 PMC6730231

[B30] Massimini M. Huber R. Ferrarelli F. Hill S. Tononi G. (2004). The sleep slow oscillation as a traveling wave. J. Neurosci. 24, 6862–6870. 10.1523/JNEUROSCI.1318-04.2004 15295020 PMC6729597

[B31] Ngo H.-V. V. Martinetz T. Born J. Mölle M. (2013). Auditory closed-loop stimulation of the sleep slow oscillation enhances memory. Neuron 78, 545–553. 10.1016/j.neuron.2013.03.006 23583623

[B32] Ngo H.-V. V. Miedema A. Faude I. Martinetz T. Mölle M. Born J. (2015). Driving sleep slow oscillations by auditory closed-loop stimulation—a self-limiting process. J. Neurosci. 35, 6630–6638. 10.1523/JNEUROSCI.3133-14.2015 25926443 PMC4412888

[B33] O'Hara R. Brooks J. O. Friedman L. Schröder C. M. Morgan K. S. Kraemer H. C. (2007). Long-term effects of mnemonic training in community-dwelling older adults. J. Psychiatr. Res. 41, 585–590. 10.1016/j.jpsychires.2006.04.010 16780878

[B34] O'Hara R. Yesavage J. A. Kraemer H. C. Mauricio M. Friedman L. F. Murphy G. M. (1998). The APOE? 4 allele is associated with decline on delayed recall performance in community-dwelling older adults. J. Am. Geriatr. Soc. 46, 1493–1498. 10.1111/j.1532-5415.1998.tb01532.x 9848808

[B35] Ohayon M. M. Carskadon M. A. Guilleminault C. Vitiello M. V. (2004). Meta-analysis of quantitative sleep parameters from childhood to old age in healthy individuals: developing normative sleep values across the human lifespan. Sleep 27, 1255–1273. 10.1093/sleep/27.7.1255 15586779

[B36] Ong J. L. Patanaik A. Chee N. I. Lee X. K. Poh J.-H. Chee M. W. (2018). Auditory stimulation of sleep slow oscillations modulates subsequent memory encoding through altered hippocampal function. Sleep 41:zsy031. 10.1093/sleep/zsy031 29425369 PMC5946855

[B37] Papalambros N. A. Santostasi G. Malkani R. G. Braun R. Weintraub S. Paller K. A.. (2017). Acoustic enhancement of sleep slow oscillations and concomitant memory improvement in older adults. Front. Hum. Neurosci. 11:109. 10.3389/fnhum.2017.00109 28337134 PMC5340797

[B38] Peigneux P. Laureys S. Fuchs S. Collette F. Perrin F. Reggers J.. (2004). Are spatial memories strengthened in the human hippocampus during slow wave sleep? Neuron 44, 535–545. 10.1016/j.neuron.2004.10.007 15504332

[B39] Plihal W. Born J. (1997). Effects of early and late nocturnal sleep on declarative and procedural memory. J. Cogn. Neurosci. 9, 534–547. 10.1162/jocn.1997.9.4.534 23968216

[B40] Rasch B. Born J. (2007). Maintaining memories by reactivation. Curr. Opin. Neurobiol. 17, 698–703. 10.1016/j.conb.2007.11.007 18222688

[B41] Rasch B. Büchel C. Gais S. Born J. (2007). Odor cues during slow-wave sleep prompt declarative memory consolidation. Science 315, 1426–1429. 10.1126/science.1138581 17347444

[B42] Rauchs G. Schabus M. Parapatics S. Bertran F. Clochon P. Hot P.. (2008). Is there a link between sleep changes and memory in Alzheimer's disease? Neuroreport 19:1159. 10.1097/WNR.0b013e32830867c4 18596620 PMC2925139

[B43] Rechtschaffen A. Kales A. (1968). A Manual of Standardized Terminology, Techniques, and Scoring System for Sleep Stages of Human Subjects. Los Angeles, CA: UCLS Brain Information Service.

[B44] Redline S. Kirchner H. L. Quan S. F. Gottlieb D. J. Kapur V. Newman A. (2004). The effects of age, sex, ethnicity, and sleep-disordered breathing on sleep architecture. Arch. Intern. Med. 164, 406–418. 10.1001/archinte.164.4.406 14980992

[B45] Ruehland W. R. O'Donoghue F. J. Pierce R. J. Thornton A. T. Singh P. Copland J. M.. (2011). The 2007 AASM recommendations for EEG electrode placement in polysomnography: impact on sleep and cortical arousal scoring. Sleep 34, 73–81. 10.1093/sleep/34.1.73 21203376 PMC3001799

[B46] Scullin M. K. (2013). Sleep, memory, and aging: the link between slow-wave sleep and episodic memory changes from younger to older adults. Psychol. Aging 28, 105–114. 10.1037/a0028830 22708533 PMC3532961

[B47] Scullin M. K. Bliwise D. L. (2015). Sleep, cognition, and normal aging: integrating a half century of multidisciplinary research. Perspect. Psychol. Sci. 10, 97–137. 10.1177/1745691614556680 25620997 PMC4302758

[B48] Shu Y. Hasenstaub A. McCormick D. A. (2003). Turning on and off recurrent balanced cortical activity. Nature 423:288. 10.1038/nature01616 12748642

[B49] Song Y. Blackwell T. Yaffe K. Ancoli-Israel S. Redline S. Stone K. L.. (2015). Relationships between sleep stages and changes in cognitive function in older men: the MrOS Sleep Study. Sleep 38, 411–421. 10.5665/sleep.4500 25325465 PMC4335525

[B50] Spiegel R. Herzog A. Köberle S. (1999). Polygraphic sleep criteria as predictors of successful aging: an exploratory longitudinal study. Biol. Psychiatry 45, 435–442. 10.1016/S0006-3223(98)00042-0 10071714

[B51] Sprecher K. E. Riedner B. A. Smith R. F. Tononi G. Davidson R. J. Benca R. M. (2016). High resolution topography of age-related changes in non-rapid eye movement sleep electroencephalography. PLoS ONE 11:e0149770. 10.1371/journal.pone.0149770 26901503 PMC4764685

[B52] Steriade M. Nunez A. Amzica F. (1993). A novel slow (<1 Hz) oscillation of neocortical neurons in vivo: depolarizing and hyperpolarizing components. J. Neurosci. 13, 3252–3265. 10.1523/JNEUROSCI.13-08-03252.1993 8340806 PMC6576541

[B53] Takashima A. Petersson K. M. Rutters F. Tendolkar I. Jensen O. Zwarts M.. (2006). Declarative memory consolidation in humans: a prospective functional magnetic resonance imaging study. Proc. Nat. Acad. Sci U. S. A. 103, 756–761. 10.1073/pnas.0507774103 16407110 PMC1334654

[B54] Timofeev I. Grenier F. Bazhenov M. Sejnowski T. Steriade M. (2000). Origin of slow cortical oscillations in deafferented cortical slabs. Cereb. Cortex 10, 1185–1199. 10.1093/cercor/10.12.1185 11073868

[B55] Timofeev I. Steriade M. (1996). Low-frequency rhythms in the thalamus of intact-cortex and decorticated cats. J. Neurophysiol. 76, 4152–4168. 10.1152/jn.1996.76.6.4152 8985908

[B56] Tucker M. A. Hirota Y. Wamsley E. J. Lau H. Chaklader A. Fishbein W. (2006). A daytime nap containing solely non-REM sleep enhances declarative but not procedural memory. Neurobiol. Learn. Mem. 86, 241–247. 10.1016/j.nlm.2006.03.005 16647282

[B57] Varga A. W. Ducca E. L. Kishi A. Fischer E. Parekh A. Koushyk V.. (2016). Effects of aging on slow-wave sleep dynamics and human spatial navigational memory consolidation. Neurobiol. Aging 42, 142–149. 10.1016/j.neurobiolaging.2016.03.008 27143431 PMC4857208

[B58] Vienne J. Lecciso G. Constantinescu I. Schwartz S. Franken P. Heinzer R.. (2012). Differential effects of sodium oxybate and baclofen on EEG, sleep, neurobehavioral performance, and memory. Sleep 35, 1071–1084. 10.5665/sleep.1992 22851803 PMC3397788

[B59] Walker M. P. (2009). The role of slow wave sleep in memory processing. J. Clin. Sleep Med. 5, 20–26. 10.5664/jcsm.5.2S.S20 19998871 PMC2824214

[B60] Walker M. P. Brakefield T. Morgan A. Hobson J. A. Stickgold R. (2002). Practice with sleep makes perfect: sleep-dependent motor skill learning. Neuron 35, 205–211. 10.1016/S0896-6273(02)00746-8 12123620

[B61] Walker M. P. Brakefield T. Seidman J. Morgan A. Hobson J. A. Stickgold R. (2003). Sleep and the time course of motor skill learning. Learn. Memory 10, 275–284. 10.1101/lm.58503 12888546 PMC202318

[B62] Walker M. P. Stickgold R. (2010). Overnight alchemy: sleep-dependent memory evolution. Nat. Rev. Neurosci. 11:218. 10.1038/nrn2762-c1 20168316 PMC2891532

[B63] Walsh J. K. Hall-Porter J. M. Griffin K. S. Dodson E. R. Forst E. H. Curry D. T.. (2010). Enhancing slow wave sleep with sodium oxybate reduces the behavioral and physiological impact of sleep loss. Sleep 33, 1217–1225. 10.1093/sleep/33.9.1217 20857869 PMC2938863

[B64] Weaver T. E. Laizner A. M. Evans L. K. Maislin G. Chugh D. K. Lyon K.. (1997). An instrument to measure functional status outcomes for disorders of excessive sleepiness. Sleep 20, 835–843. 9415942

[B65] Welch P. (1967). The use of fast Fourier transform for the estimation of power spectra: a method based on time averaging over short, modified periodograms. IEEE Trans. Audio Electroacoust. 15, 70–73. 10.1109/TAU.1967.1161901

[B66] Westerberg C. E. Florczak S. M. Weintraub S. Mesulam M.-M. Marshall L. Zee P. C.. (2015). Memory improvement via slow-oscillatory stimulation during sleep in older adults. Neurobiol. Aging 36, 2577–2586. 10.1016/j.neurobiolaging.2015.05.014 26116933 PMC4523433

[B67] Westerberg C. E. Lundgren E. M. Florczak S. M. Mesulam M.-M. Weintraub S. Zee P. C.. (2010). Sleep influences the severity of memory disruption in amnestic mild cognitive impairment: results from sleep self-assessment and continuous activity monitoring. Alzheimer Dis. Assoc. Disord. 24:325. 10.1097/WAD.0b013e3181e30846 20592579 PMC3025089

[B68] Westerberg C. E. Mander B. A. Florczak S. M. Weintraub S. Mesulam M.-M. Zee P. C.. (2012). Concurrent impairments in sleep and memory in amnestic mild cognitive impairment. J. Int. Neuropsychol. Soc. 18:490. 10.1017/S135561771200001X 22300710 PMC3468412

[B69] Wilson J. K. Baran B. Pace-Schott E. F. Ivry R. B. Spencer R. M. (2012). Sleep modulates word-pair learning but not motor sequence learning in healthy older adults. Neurobiol. Aging 33, 991–1000. 10.1016/j.neurobiolaging.2011.06.029 22244090 PMC3307877

[B70] Wilson M. A. McNaughton B. L. (1994). Reactivation of hippocampal ensemble memories during sleep. Science 265, 676–679. 10.1126/science.8036517 8036517

[B71] Yesavage J. A. Sheikh J. I. Friedman L. Tanke E. (1990). Learning mnemonics: roles of aging and subtle cognitive impairment. Psychol. Aging 5:133. 10.1037/0882-7974.5.1.133 2317292

[B72] Zhang Y. Gruber R. (2019). Focus: attention science: can slow-wave sleep enhancement improve memory? A review of current approaches and cognitive outcomes. Yale J. Biol. Med. 92:63. 30923474 PMC6430170

