# Supplementary material for: High-Resolution Spectral Sleep Analysis Reveals a Novel Association Between Slow Oscillations and Memory Retention in Elderly Adults
Source: Front Aging Neurosci. 2021 Jan 11;12:540424. doi: 10.3389/fnagi.2020.540424 (PMC7829345; doi:10.3389/fnagi.2020.540424)
Supplement: Supplementary file 1 [file Table_1.DOCX]

Supplement Table 1: Partial correlational analysis with SWS in 50 participants who have a higher recall score in the evening.

| 50 participants scored higher in Evening 5-min recall, partial correlation with SWS, covaried for age, gender, years of education | | | |
| --- | --- | --- | --- |
|  | Mean and SD | Correlation | p |
| Evening 5-min recall List A | 9.7±2.25 | 0.224 | 0.134 |
| Overnight morning recall List A | 7.4±3.2 | 0.357 | 0.015 |
| Morning 5-min recall with alt List B | 9.1±3.6 | 0.24 | 0.109 |
| OMR | 0.75±0.24 | 0.313 | 0.034 |

SD: Standard deviation

SWS: slow wave sleep (%)

Evening 5-min recall List A: Pre-sleep evening delayed recall List A total score prior to PSG

Overnight morning recall List A: Post-sleep/following-morning, delayed-recall performance on List A total score

Morning 5-min recall alt List B: Post-sleep/morning-administered, delayed-recall performance on alternate List B total score

OMR: Overnight memory retention, defined as post-sleep/following-morning, delayed-recall performance on List A total score divided by pre-sleep evening delayed-recall performance on List A total score

Supplement Table 2: Partial correlational analysis covaried for age, gender, and years of education, in all participants to examine the association of slow oscillation (0.5-1 Hz) relative power during the first 88.5 minutes of sleep and list-learning scores in all 101 participants and correlation coefficient.

| All 101 participants, partial correlational analysis with slow oscillation relative power during the first 88.5 min (177 epochs), covaried for age, gender, and years of education | | |
| --- | --- | --- |
|  | Correlation | p |
| Evening 5-min recall List A | 0.080 | 0.436 |
| Overnight morning recall List A | 0.178 | 0.081 |
| Morning 5-min recall with alt List B | 0.070 | 0.494 |
| OMR ratio | 0.218 | 0.032 |

Supplement Table 3: Spearman's bivariate correlation analysis of the relative power spectrum of slow oscillation without adjustment for covariates in all participants.

| All 101 participants, correlational analysis with relative power spectrum of slow oscillaition during the first 88.5 min (177 epochs), without adjustment for covariates | | |
| --- | --- | --- |
|  | Correlation | p |
| Evening 5-min recall List A | 0.000 | 0.998 |
| Overnight morning recall List A | 0.113 | 0.260 |
| Morning 5-min recall with alt List B | -0.009 | 0.926 |
| OMRratio | 0.167 | 0.095 |

Evening 5-min recall List A: Pre-sleep evening delayed recall List A total score prior to PSG

Overnight morning recall List A: Post-sleep/following-morning, delayed-recall performance on List A total score

Morning 5-min recall alt List B: Post-sleep/morning-administered, delayed-recall performance on alternate List B total score

OMR: Overnight memory retention, defined as post-sleep/following-morning, delayed-recall performance on List A total score divided by pre-sleep evening delayed-recall performance on List A total score

Supplement Table 4: Spearman's bivariate correlation analysis of relative power spectrum of slow oscillation without adjustment for covariates in SWS (-) group

| 77 participants without SWS, correlational analysis with relative power spectrum of slow oscillaition during the first 88.5 min (177 epochs), without adjustment for covariates | | |
| --- | --- | --- |
| Dependent variables | Correlation | p |
| Evening 5-min recall List A | 0.026 | 0.820 |
| Overnight morning recall List A | 0.212 | 0.065 |
| Morning 5-min recall with alt List B | -0.043 | 0.710 |
| OMR | 0.299 | **0.008*** |

Evening 5-min recall List A: Pre-sleep evening delayed recall List A total score prior to PSG

Overnight morning recall List A: Post-sleep/following-morning, delayed-recall performance on List A total score

Morning 5-min recall alt List B: Post-sleep/morning-administered, delayed-recall performance on alternate List B total score

OMR: Overnight memory retention, defined as post-sleep/following-morning, delayed-recall performance on List A total score divided by pre-sleep evening delayed-recall performance on List A total score

Supplemental Table 5

|  |  | SWS (-) | SWS (+) |  |
| --- | --- | --- | --- | --- |
| Demographics | Gender | Women=37, Men=40 | Women=16, Men=8 |  |
|  | Age | 71.3±8.1 | 69.2±7.1 | years |
|  | Years of Education | 16.7±2.5 | 16.6±3.3 | years |
|  | BMI | 27.5±4.9 | 28.7±4.3 | kg/m2 |
| PSG parameters | TST | 350.1±76.3 | 325.7±92.1 | min |
|  | TIB | 510.1±84.3 | 508.1±111.3 | min |
|  | N1 | 17.8±10.4 | 13.6±9.2 | % |
|  | N2 | 69.5±11.1 | 66.7±12.5 | % |
|  | N3 (=SWS) |  | 5.7±5.0 | % |
|  | REM | 12.7±4.9 | 14±8.4 | % |
|  | SL | 40.8±49 | 28.3±31.9 | min |
|  | WASO | 123.4±74.7 | 132±96 | min |
|  | SE | 69.5±14.5 | 65.7±18.4 | % |
|  | AHI | 12.1±12.5 | 9.6±14.3 | events/hour |
|  | AvSpO2 | 92.9±10.8 | 93.8±2.2 | % |
|  | MinSpO2 | 78.2±21.3 | 84.3±5 | % |
|  | PLMI | 8.7±15.9 | 7.1±8.3 | events/hour |
| Sleep questionnaires | ESS | 7.4±3.8 | 7.3±5.6 |  |
|  | PSQI | 6.9±3.9 | 7.1±4 |  |
|  | FOSQ | 17.9±2.2 | 17.8±3 |  |
|  | MEQ | 59.8±9.0 | 55.3±7.4 |  |

Numbers represent mean ± standard deviation, except gender, which showed the number of participants. BMI: body mass index (kg/m^2^), TST: total sleep time (minutes), TIB: time in bed (minutes), N1 to N3: Stage N1 to N3 sleep (%), SWS: slow wave sleep, REM: rapid eye movement sleep (%), SL: sleep latency (minutes) , WASO: wake after sleep onset (minutes), SE: sleep efficiency (%), AHI: apnea hypopnea index (events/hour), AvSpO2: average oxygen saturation (%), MinSpO2: minimum oxygen saturation (%), PLMI: periodic limb movement index (events/hour), ESS: Epworth Sleepiness Scale, PSQI: Pittsburgh Sleep Quality Index, FOSQ: Functional Outcomes of Sleep Questionnaire, MEQ: Morningness-Eveningness Questionnaire.

Supplement Table 6

| All 101 participants, partial correlational analysis with slow oscillation relative power during the first 88.5 min (177 epochs), covaried for age, gender, and years of education | | |
| --- | --- | --- |
|  | Correlation | p |
| Evening 5-min recall List A | 0.080 | 0.436 |
| Overnight morning recall List A | 0.178 | 0.081 |
| Morning 5-min recall with alt List B | 0.070 | 0.494 |
| OMR ratio | 0.218 | 0.032 |
| OMR subtraction | -0.172 | 0.092 |

Evening 5-min recall List A: Pre-sleep evening delayed recall List A total score prior to PSG

Overnight morning recall List A: Post-sleep/following-morning, delayed-recall performance on List A total score

Morning 5-min recall alt List B: Post-sleep/morning-administered, delayed-recall performance on alternate List B total score

OMR ratio: Overnight memory retention, defined as post-sleep/following-morning, delayed-recall performance on List A total score divided by pre-sleep evening delayed-recall performance on List A total score

OMR subtraction: Overnight memory retention, defined as the subtraction of post-sleep/following-morning, delayed-recall performance on List A total score from pre-sleep evening delayed-recall performance on List A total score.

Supplement Table 7

| 77 participants without SWS, partial correlation with relative power spectrum of slow oscillaition during the first 88.5 min (177 epochs), covaried for age, gender, years of education | | |
| --- | --- | --- |
|  | Correlation | p |
| Evening 5-min recall List A | 0.092 | 0.434 |
| Overnight morning recall List A | 0.257 | 0.027 |
| Morning 5-min recall with alt List B | 0.032 | 0.789 |
| OMR ratio | 0.335 | **0.003*** |
| OMR subtraction | -0.278 | 0.016 |

Evening 5-min recall List A: Pre-sleep evening delayed recall List A total score prior to PSG

Overnight morning recall List A: Post-sleep/following-morning, delayed-recall performance on List A total score

Morning 5-min recall alt List B: Post-sleep/morning-administered, delayed-recall performance on alternate List B total score

OMR ratio: Overnight memory retention, defined as post-sleep/following-morning, delayed-recall performance on List A total score divided by pre-sleep evening delayed-recall performance on List A total score

OMR subtraction: Overnight memory retention, defined as the subtraction of post-sleep/following-morning, delayed-recall performance on List A total score from pre-sleep evening delayed-recall performance on List A total score.
